# Aluminum Perlite Syntactic Foams

**DOI:** 10.3390/ma15155446

**Published:** 2022-08-08

**Authors:** György Thalmaier, Niculina Argentina Sechel, Alexandra Csapai, Catalin Ovidiu Popa, Gabriel Batin, Andras Gábora, Tamas Mankovits, Ioan Vida-Simiti

**Affiliations:** 1 Department of Materials Science and Engineering, Technical University of Cluj-Napoca, 103-105 Muncii Ave., 400641 Cluj-Napoca, Romania; 2 Department of Mechanical Engineering, Faculty of Engineering, University of Debrecen, Ótemető u. 2-4, H-4028 Debrecen, Hungary; 3 Technical Science Academy of Romania, Dacia Ave., 26, 010413 Bucharest, Romania

**Keywords:** syntactic foams, spark plasma sintering, expanded perlite, compression tests

## Abstract

This paper presents the usage of spark plasma sintering (SPS) as a method to obtain aluminum-expanded perlite syntactic foams with high porosity. In the test samples, fine aluminum powder with flaky shape particles was used as matrix material and natural, inorganic, granular, expanded perlite was used as a space holder to ensure high porosity (35–57%) and uniform structure. SPS was used to consolidate the specimens. The structures were characterized by scanning electron microscopy and compression tests. Energy absorption (W~7.49 MJ/m^3^) and energy absorption efficiency (EW < 90%) were also determined.

## 1. Introduction

Porous materials became an interest matter for researchers both in the industrial and scientific field due their combination of unique physical, mechanical, thermal, electrical, and acoustic properties in conjunction with low density and high specific strength [1,2,3,4,5]. The idea that inspired the creation of these cellular materials finds its roots in natural elements such as wood, pumice stone, bones and other materials which have been used for different applications for a long period of time [6]. The first reference to cellular metals or metallic foams can be found in a French patent published in 1925, where a foamed precursor to create an expanded structure with high porosity was used for the fabrication process. Interest in the matter has slowly increased until the mid-1990s, when the number of published articles about metallic foams increased significantly, by 20% annually [7]. Although the number of research papers and reports has increased for porous metals and related structures overall, aluminum, nickel and titanium continue to be the area of focus for the subject [8].

The selection of the materials is based on the end application requirements and economic justification [9]. There are nine distinct process-routes developed to make metal foams, from which five are used commercially. These processes can be classified into four broad classes: foams formed from the vapor phase, which includes the vapor deposition technique; foams electrodeposited from an aqueous solution or electrochemical deposition; foams obtained by liquid-state processing, which includes techniques such as direct foaming with gas, direct foaming with blowing agents, gasars, powder compact melting, casting and spray foaming; foams created in the solid state, which comprises the following methods: sintering of hollow spheres, gas entrapment, slurry foaming, pressing around fillers, sintering of powders or fibers, extrusion of polymer/metal mixtures and reaction sintering [10,11] and pore size as low as tens of nanometers [12].

Syntactic foams represent a class of closed cell foams synthetized by dispersing rigid hollow particles in a matrix material [13,14,15,16,17]. Classical processing techniques such as gas bubbling in molten metal shortfall in control over the size and the structure of the pore walls, as well as the percentage of porosity within the material. In comparison, powder sintering techniques allow control over the size of the pores. One such technique is spark plasma sintering (SPS), which uses the action of pulsed direct current, providing adaptability regarding the heating rate and heating mode, thus ensuring control over the microstructure, phase composition and structure of the pore walls [18].

Thanks to their internal structure, which can be porous, cellular or filled with hollow particles, syntactic foams show excellent mechanical properties such as compressive strength combined with good energy absorption and light weight [19,20,21,22]. Considering these properties and the possibility of tailoring them, various applications can be designed in industries such as automotive, aeronautics, constructions [23,24], biomaterials [21,25] and defense [20].

To achieve the desired properties various combinations of matrix and spacers were proposed. Abdullah et al. [21] suggested a combination between SS316L and carbamide for biomaterial applications, while Jain et. al. [25] used 316L/urea particles for the same purpose. Low carbon steel AISI 1018 and hollow alumina microspheres were studied by Castro and Nutt [26] for energy absorption applications. Aluminum is by far the material most used as matrix with various space holders; Al7075-T/ceramic spheres [20], Al-7075/pumice particles [24], Al1050/globocer particles and A413.0/globocer [27], Al/soda-lime glass particles [28], A356/perlite [29], Al/expanded perlite [30], 2014Al/cenosphere particles [31], Al/Mg [23]. Zinc alloy ZA27 filled with expanded perlite was studied by Movahedi and his collaborators [32]. Magnesium is another metal studied to produce foams, such as AZ91/activated carbon in [33], and AZ91/Ni-P coated fly ash [34]. Other researchers reported their results about the titanium foams. Jha and coauthors produced titanium syntactic foam with coarser cenosphere by powder metallurgy [35]. Xue and Zhao investigated in [36] the obtaining of titanium foam with ceramic microspheres for implants.

Most of the up-to-date research on the syntactic metallic foams rely on the melt infiltration to manufacture the desired foams. The present paper investigates an alternative to manufacture these foams based on a powder metallurgy approach. This method allows to manufacture foams which are difficult to process or need special care during manufacturing.

## 2. Materials and Methods

**Figure 1:**
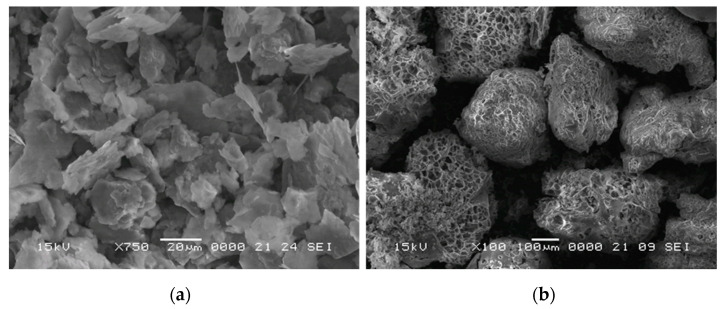
SEM images of the (**a**) raw aluminum powder and (**b**) expanded perlite particles.

In the present study, the raw materials were fine aluminum powder (obtained by mechanical milling) and commercially available (Australian Perlite Pty) expanded perlite, a natural, inorganic, granular material. The main advantage of using perlite particles is the ease of controlling the pore size of closed cell metallic foams compared to the other available options. The aluminum powder particles have a flake-like shape, with a size distribution of 20–40 μm and thickness of ~0.5 μm (Figure 1a). The aluminum powder was characterized using scanning electron microscopy (SEM). The same Jeol JSM-5600 LV scanning electron microscope equipped with an EDS probe (Oxford Instruments) was used to characterize the expanded perlite particles. The SEM image in Figure 1b shows pearlite particles that were completely expanded, with a porous, honeycomb-like internal structure and a sieving diameter < 500 µm, while the aspect ratio was about 0.6.

**Figure 2:**
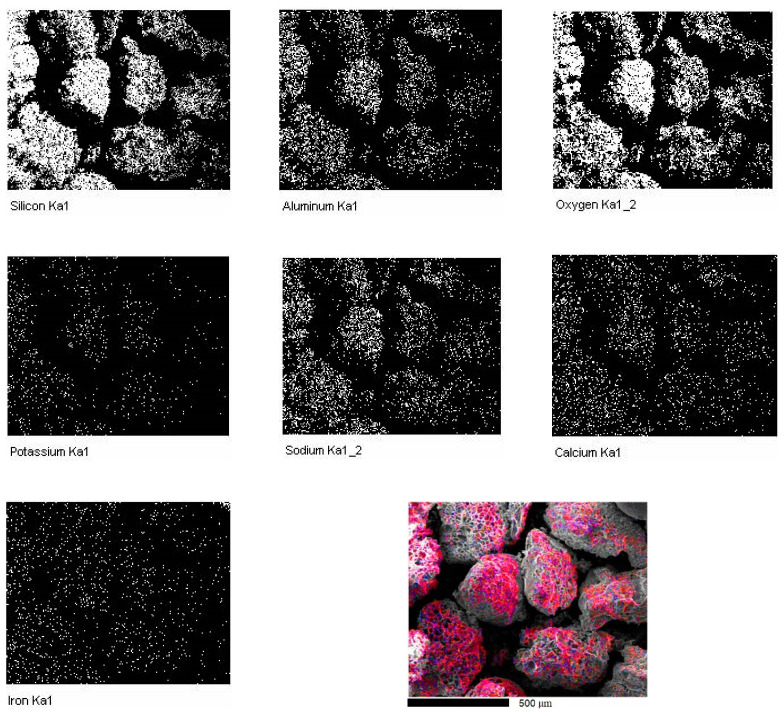
EDX analysis showing the distribution map of the present elements in the perlite particles and overlapping map of the detected elements by EDX- analysis.

The expanded perlite particles have in the composition according to the material data sheet mainly SiO_2_ and Al_2_O_3_ (75 wt.% SiO_2_, 14 wt.% Al_2_O_3_, 4 wt.% K_2_O, 3 wt.% Na_2_O, 1.3 wt.% CaO, 1 wt.% Fe_2_O_3_ and some minor traces of other oxides), with traces of other oxides, as also suggested by the EDX analysis, presented in Figure 2.

**Figure 3:**
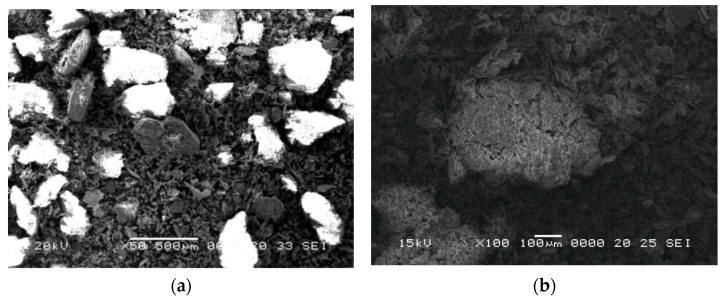
SEM image of the mixture of aluminum powder and perlite particles (**a**) and aluminum coated perlite particle (**b**) for the mixture containing 60% perlite particles.

After 15 min of mixing, the particles were fully mixed. The homogenous blend is shown by the SEM images in Figure 3. No specific measures were taken regarding the atmosphere in which the aluminum powder and the perlite particles were homogenized.

Different volume fractions (60%, 70% and 80%) of perlite powder were mixed with the aluminum powder and homogenized for 15 min in a Turbula type spatial mixer. During this step of the process, the aluminum powder covers the perlite particles (Figure 3b), forming a thin layer around them which improves the sinterability of the samples. The required perlite weight fractions were calculated using the following formula:

Weight% = (x∙ρ_perlite_)/(ρ_Al_∙(100 − x)) (1)

where x = volume fraction of the perlite, ρ_perlite_ = the density of perlite (0.2 g/cm^3^), ρ_Al_ = the density of aluminum (2.71 g/cm^3^).

The mixtures were sintered by SPS on a “homemade” SPS system using carbon die and punches. The sintering process was performed in argon atmosphere, at 550 °C and held for 30 s at the maximum sintering temperature, as established by preliminary tests. A pressure of 10 MPa was maintained during the sintering process. Samples with a diameter of 10.5 mm and a height of approximately 10.5 mm were obtained. The density of the samples was calculated by measuring their dimensions with a 0.05 mm precision using a caliper and dividing the mass by the calculated cylindrical volume. The samples’ porosity was estimated by calculating the mixtures density and subsequently calculating porosity using Equation (2).

P = 1 − ρ_s_/ρ_m_ (2)

where P is the porosity, ρ_s_ is the sample’s density and ρ_m_ is the aluminum-expanded perlite mixture’s theoretical density. The calculated porosity is composed of “large” pores generated by the added perlite particles and small pores as voids between the aluminum particles, that are specific to powder metallurgy techniques. The microtomography analysis of the samples (µCT) was performed on a Brucker-microCT SkyScan 1172 system. The Materialize Mimics software was used in the reconstruction process. The porous particles were measured again by image analysis on the reconstructed samples using ImageJ software.

The mechanical behavior of the obtained foams at room temperature under compressive load was studied through experimental tests (3 tests for each sample type). These tests were performed on a ZwickRoell Z005 testing machine with a test speed of 0.2 mm/min, on samples with a diameter of 10.5 mm and a D/H ratio~1.

The samples containing 60% expanded perlite did not have a “foam-like” compression behavior so only the last two compositions are evaluated from this point of view. The energy absorption was calculated by integrating the area below the compression up to a deformation of 30% curve for the samples containing 70% and 80% volume percentage of expanded perlite particles. The energy absorption efficiency was calculated by dividing the energy absorption to the product of the maximum compressive stress within the strain range and the magnitude of the strain range as specified by the ISO-13314 standard.

## 3. Results and Discussions

### 3.1. The Sintered Samples

The main concern when processing aluminum foams from solid state through classical powder metallurgy methods is the oxide layer that forms around the surface of the aluminum particles.

The oxide layer hinders diffusion between particles and therefore the formation of solid sintering necks. To have better control over the sintering process and parameters, spark plasma sintering was applied. This method ensures the capacity of removing the oxide layers, providing a better bonding between the particles. Another advantage provided by the SPS method is the ability to control and decrease the sintering time and temperature, compared to classical sintering. This leads to a fine-grained microstructure within the aluminum foam due to the limited grain growth during the sintering stage. 

**Figure 4:**
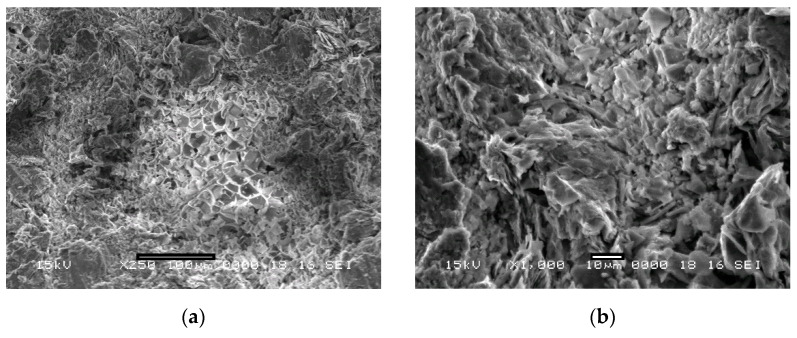

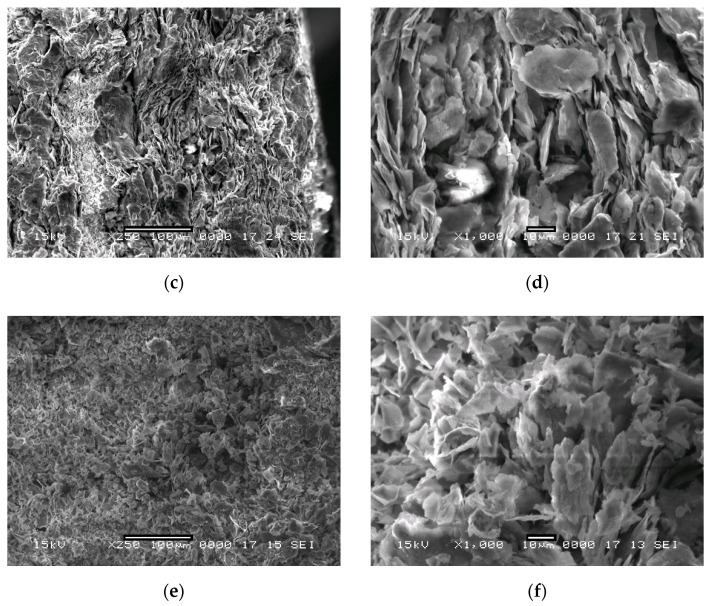
Low and high magnification SEM images of a section through: (**a**,**b**) sample with 60% volume percentage of expanded perlite; (**c**,**d**) sample with 70% volume percentage of expanded perlite; (**e**,**f**) sample with 80% volume percentage of expanded perlite.

The density of the samples was calculated by dividing the mass of the samples after sintering by the calculated volume and the following results were obtained (Table 1):

The morphology of the samples was analyzed by scanning electron microscopy (Figure 4). The SEM images show a well sintered structure for the three types of samples, with a good bond between the aluminum particles, the perlite particles act only as a pore former.

In Figure 4a, the honeycomb-like structure of the expanded perlite particle embedded in the aluminum matrix is visible. This indicates a good sintering and a good consolidation between the matrix and the expanded perlite. Similarly, for Figure 4b,c, both the samples containing 70% and 80% volume fraction of expanded perlite suggest a uniform sintering, a well-defined, coherent surface, indicating a stable, well- reinforced structure.

### 3.2. Samples 3D Structure

**Figure 5:**
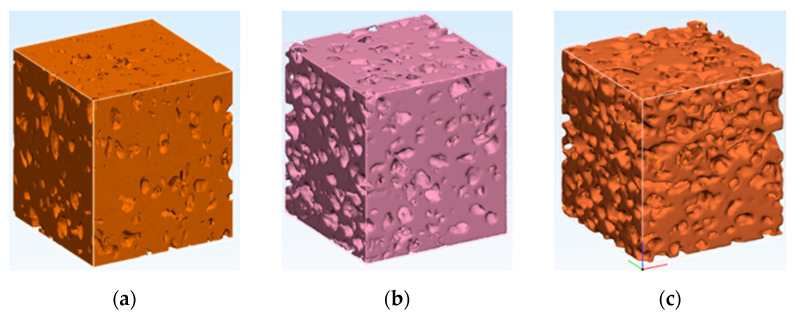
Image analysis of particle sizes on the reconstructed samples of (**a**) Sample 1, 60% volume percentage of expanded perlite, (**b**) Sample 2, 70% volume percentage of expanded perlite, (**c**) Sample 3, 80% volume percentage of expanded perlite.

The reconstruction procedure was based on a series of µCT images saved in tiff format. Figure 5 presents the µCT images of cubes having a side of 3.4 mm from the samples. All samples presented a good homogeneity. For each sample, the porosity was measured in six perpendicular planes by image analysis. The standard deviation on the porosity measurements was ~10%.

The reconstruction process of the samples after the µCT analysis were conducted so that the expanded perlite’s places is shown as a pore. The aim of this was to analyze the influence of the pressure on the final structure. The use of pressure during sintering ensures a good electrical contact between the particles. If the pressure is too high, the porous particles are crushed, and an increased densification will occur.

**Figure 6:**
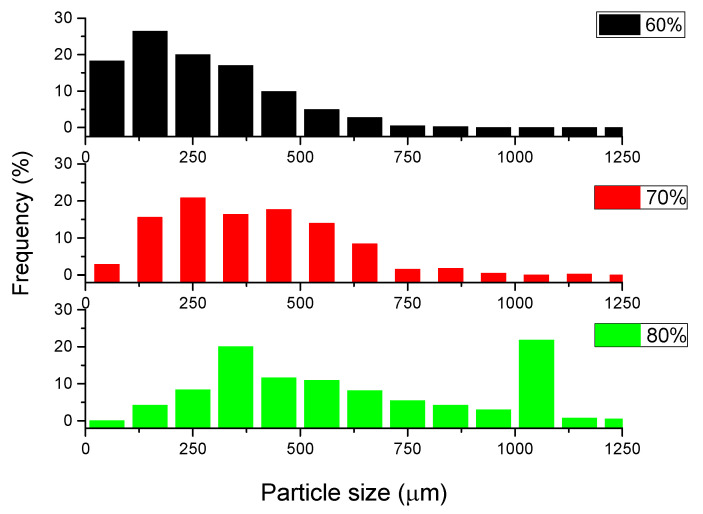
Perlite particles distribution within the sintered samples with 60, 70 and 80% expanded perlite particles.

As presented in Figure 5 and Figure 6, there is a good agreement between the particle size of the porous particles and the results of the reconstruction process. Some particles were pressed together during the SPS process and could not be separated in the µCT analysis. These measurements assured that, in the limits of the experimental errors, the initial porous structure was not negatively influenced by the applied pressure.

### 3.3. Compression Test Results

**Figure 7:**
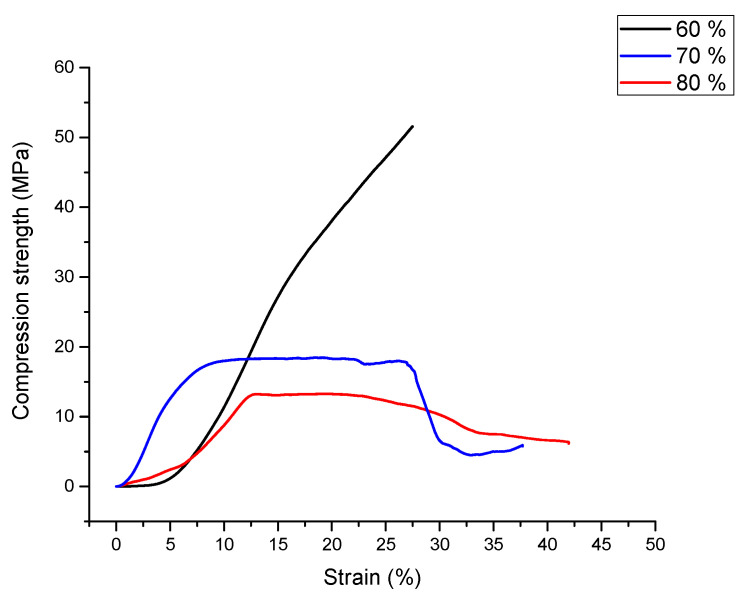
Compression curves for samples with 60, 70 and 80% expanded perlite.

Metal foams can be well controllable in mechanical properties by means of the porosity [24]. Typical compression curves of the aluminum-expanded perlite syntactic foams are shown in Figure 7. This figure displays different compressive behaviors of the syntactic foam samples with different porosities. The compressive stress-strain curve of the first specimen, containing 60% perlite, shows an abrupt slope, with a consistent and constant increase in stress. The typical plastic deformation plateau, specific to metallic foams, is not present. The shift in the curvature can be considered as the compression strength according to Ashby [11]. The compression behavior is similar to the deformation curve of the aluminum matrix. This type of deformation behavior was observed in other aluminum alloys as well [30].

Both curves for samples containing 70% and 80% vol. expanded perlite are similar to the typical compression curves of syntactic foams [29,30,37]. Both curves present a local maximum, depicting the compression stress and the typical plastic deformation plateau. The higher values of compression and plateau strength for a content of 70 vol% expanded perlite can be attributed to a higher value of density comparative with samples having 60 vol% of filler. A higher density means a higher content of matrix. A lower content of expanded pearlite will increase the compression strength but under a certain content (in this situation 60 vol% filler) will tend to cancel the foam-like behavior. For these two samples, the energy absorption (W defined as the under the compression curve up to a deformation of 30%) and energy absorption efficiency (EW representing the ratio between the absorbed energy (W) and the product between the maximum stress and the deformation at the maximum stress, considered to be the maximum absorbed energy) were determined.

The greatest energy absorption efficiency was observed for the samples containing 70% expanded perlite, as shown in Table 2. For the 70% vol. perlite samples, W = 7.49 MJ/m^3^ and the efficiency was EW = 90%, while for the 80% vol. perlite samples we obtained W = 3.22 MJ/m^3^ and EW = 70%. As the expanded perlite fraction increases, there is less and less aluminum to assure the samples’ mechanical response and to maintain a consistent and continuous metallic matrix around the ceramic particles. The abrupt failure of the sample with 70% perlite assures a high energy absorption efficiency similar to other authors’ findings [15,16,17], however, the steady decrease of the stresses by increasing strain for the higher perlite content samples reduces this efficiency considerably.

## 4. Conclusions

As a result of our study regarding the syntactic metallic foams, the followings can be concluded:

- Metallic foams are attracting increasing interest due to the unique combination of physical and mechanical properties such as low density and high specific strength.
- Syntactic metallic foams were successfully produced by Spark Plasma Sintering using aluminum and expanded perlite.
- The particle size of expanded perlite in the sintered samples measured by SEM is in accordance with those resulted by image reconstruction of structures based on μCT. The results confirm that the pressure applied during the Spark Plasma Sintering was enough for good sinterability, preserving at the same time the filler particles.
- Samples with a content of 70, 80 vol% show the specific behavior of syntactic foams to compressive loadings, while those having 60 vol% behave similarly to the bulk aluminum.
- Compression and plateau strengths are in the range reported in literature for this type of materials.
- The best results were obtained for a fraction of 70 vol% expanded perlite, with a value of absorbed energy of 7.49 MJ/m^3^ and an efficiency of 90%.

## Figures and Tables

**Table 1:** Density of the samples based on the percentage of expanded perlite.

Perlite Amount (%vol.)	Density (g/cm^3^)	Porosity (%)
60	1.73	35.9
70	1.23	54.4
80	1.16	57.0

**Table 2:** Energy absorption and energy absorption efficiency for the samples containing 70% and 80% expanded perlite particles.

Perlite Amount (%vol.)	Compression Strength (MPa)	Plateau Strength (MPa)	Absorbed Energy (MJ/m^3^)	Energy Absorption Efficiency (%)
70	18	18	7.49	90
80	12	12.5	3.22	70

## Data Availability

Not applicable.
